# Clinical variables associated with major adverse cardiac events following radical cystectomy

**DOI:** 10.1002/bco2.315

**Published:** 2023-12-05

**Authors:** Aaron A. Gurayah, Ruben Blachman‐Braun, Christopher J. Machado, Matthew M. Mason, Helen Y. Hougen, Ali Mouzannar, Mark L. Gonzalgo, Bruno Nahar, Sanoj Punnen, Dipen J. Parekh, Chad R. Ritch

**Affiliations:** ^1^ University of Miami Miller School of Medicine Miami Florida USA; ^2^ Desai Sethi Urology Institute University of Miami Miller School of Medicine Miami Florida USA; ^3^ Sylvester Comprehensive Cancer Center Miami Florida USA

**Keywords:** age, cystectomy, diabetes mellitus, hypertension, obesity, urinary diversion

## Abstract

**Objectives:**

The objective of this study is to investigate the association between major adverse cardiac events (MACE) and clinical factors of patients undergoing radical cystectomy (RC) for bladder cancer.

**Materials and Methods:**

A retrospective analysis using the 2015–2020 National Surgical Quality Improvement Program database was performed on patients who underwent RC for bladder cancer. MACE was defined as any report of cerebrovascular accident, myocardial infarction, or thromboembolic events (pulmonary embolism or deep vein thrombosis). A multivariable‐adjusted logistic regression was conducted to identify clinical predictors of postoperative MACE.

**Results:**

A total of 10 308 (84.2%) patients underwent RC with incontinent urinary diversion (iUD), and 1938 (15.8%) underwent RC with continent urinary diversion (cUD). A total of 629 (5.1%) patients recorded a MACE, and on the multivariable‐adjusted logistic regression, it was shown that MACE was significantly associated with increased age (OR = 1.035, 95% CI: 1.024–1.046, *p* < 0.001), obesity (OR = 1.583, 95% CI: 1.266–1.978, *p* < 0.001), current smokers (OR = 1.386, 95% CI: 1.130–1.700, *p* = 0.002), congestive heart failure before surgery (OR = 1.991, 95% CI: 1.016–3.900; *p* = 0.045), hypertension (OR = 1.209, 95% CI: 1.016–1.453, *p* = 0.043), and increase the surgical time (per 10 min increase, OR = 1.010, 95% CI: 1.003–1.017, *p* = 0.009). We also report that increased age, obesity, and patients undergoing cUD (OR = 1.368, 95% CI: 1.040–1.798; *p* = 0.025) are associated with thromboembolic events.

**Conclusion:**

By considering the preoperative characteristics of patients, including age, obesity, smoking, congestive heart failure, and hypertension status, urologists may be able to decrease the incidence of MACE in patients undergoing RC. Urologists should aim for lower operative times as this was associated with a decreased risk of thromboembolic events.

## 1 INTRODUCTION

Radical cystectomy (RC) with pelvic lymphadenectomy remains the standard of therapy for muscle‐invasive bladder cancer (MIBC).^1^ Approximately 30% of patients present with MIBC, and that number increases to 40% when considering patients who progress from non‐muscle‐invasive bladder cancer (NMIBC) to MIBC.^2^, ^3^, ^4^ RC is also considered for the treatment of high‐risk and Bacillus Calmette‐Guerin unresponsive NMIBC.^5^, ^6^

RC requires the creation of a urinary diversion (UD), including incontinent urinary diversion (iUD) or continent urinary diversion (cUD). The most common type of iUD for RC is an ileal conduit urinary diversion whereas the most common cUD is an orthotopic neobladder.^7^, ^8^ Postoperative complications such as surgical site infections as well as urinary and bowel complications have been associated with both types of urinary diversions.^9^

In addition to complications resulting from UD, patients undergoing RC may also experience major adverse cardiac events (MACE) including cerebrovascular accident (CVA), myocardial infarction (MI), and thromboembolic events. Following surgery, RC patients have a reported rate of MACE ranging 1.4%–4.1% with significant postoperative morbidity and mortality.^10^ Prolonged operative time during major abdominal surgery has also been associated with an increased risk of MACE.^11^ Sakran et al. observed an association between operating room time and the development of deep vein thrombosis (DVT) or pulmonary embolus (PE); researchers identified prolonged operative time (defined as >100 min) as an independent risk factor for developing MACE. For every 10‐min increase after 100 min, the risk of developing DVT increases by 7%, and the risk of PE increases by 5%.^11^ It has also been shown that the 6‐month mortality and hospital stay are significantly greater in patients with MACE, compared to those without MACE after RC.^10^ Creation of a neobladder is technically rigorous and is typically associated with longer operative times when compared to an ileal conduit.^7^ However, in experienced hands, both cUD and iUD can be performed expeditiously.

The objective of this study was to investigate the association between MACE and clinical factors of patients undergoing RC. We hypothesized that older age, increased surgical time, and preoperative comorbidities may be associated with a higher risk of MACE. We also secondarily aimed to evaluate postoperative timing for the occurrence of MACE to determine how long these events take to develop following RC.

## 2 MATERIALS AND METHODS

### 2.1 Study design and participants selection

We queried the 2015–2020 American College of Surgeons National Surgical Quality Improvement Program (ACS NSQIP). This dataset serves as a surgical registry capturing preoperative risk factors, intraoperative variables, and 30‐day postoperative mortality and morbidity outcomes for patients undergoing major surgical procedures.^12^

Patients age ≥18 years who underwent RC, pelvic exenteration, and urinary diversion (CPT code: 51550, 51570, 51580, 51555, 51565, 51590, 51575, 51596, 51585, 51595, 51597, or 50820) were included in this study. We maintained the following exclusion criteria (Figure S1):

1. Patients without a diagnosis of bladder cancer, carcinoma in situ of the bladder, or cancer of urothelial origin (ICD‐10: C66, C65, C67, D09.0, or Z85.5, and ICD‐9: 188189, or 233.7).
2. Patients who underwent partial cystectomy (CPT code: 51550, 51 555, and 51 565), pelvic exenteration (CPT code: 51597), ileal conduit (CPT code: 50820) without associated cystectomy procedure, or ureterosigmoidostomy or ureterocutaneous diversion (CPT code: 51585 or 51 580).
3. Patients with cystectomy (CPT code: 51570 or 51 575) without associated ileal conduit or continent urinary diversion (CPT code: 51596), or unclear approach such as patients in which reported both cystectomy with continent urinary diversion and ileal conduct (CPT code: 51596 + 51580, 51596 + 51590, or 51596 + 51595).
4. Patients with reported disseminated cancer or missing clinical information.
5. Patients who had an operative time reported being <1 h or ≥±3 standard deviations from the mean operative time.

### 2.2 Variables assessment

Variables were selected from the NSQIP participant data file. BMI was categorized as underweight (<18.5 kg/m^2^), normal (18.5–24.9 kg/m^2^), overweight (25–29.9 kg/m^2^), and obese (≥30 kg/m^2^). To classify the type of urinary diversion after RC, patients that had RC with iUD were considered as those that had RC with ileal conduit or sigmoid neobladder using the following CPT codes 51590, 51595, 51570 + 50820, or 51575 + 50820, and patient that were classified as RC with cUD were those with the following CPT code 51596.

To create the variable for MACE, we considered any report of CVA, MI, or thromboembolic events (DVT or PE) as recorded in the NSQIP database. To evaluate the time from surgery to MACE in patients that reported multiple events, the episode that was first recorded was considered as the event time. To further stratify the prevalence of MACE, we divided patients based on the week of occurrence.

### 2.3 Statistical analysis

Statistical analysis was performed with SPSS version 28 (Armonk, NY). Means ± standard deviations (SD) or medians [interquartile ranges] were calculated according to the data distribution. A comparison of numerical variables between groups was performed using Student's T or Mann–Whitney *U* test as required. Categorical variables were analysed with a chi‐square test. To determine the association between MACE and clinical variables, demographics, surgical time groups, and 30‐day associated complications, a multivariable‐adjusted logistic regression analysis was performed to evaluate the risk of MACE, CVA, thromboembolic events, and MI (OR: odds ratio). A *p*‐value < 0.05 was considered statistically significant.

## 3 RESULTS

A total of 12 246 patients were analysed, consisting of 10 308 (84.2%) patients who underwent RC with iUD and 1938 (15.8%) patients who underwent RC with cUD. The mean age was 69.1 ± 9.6 years, BMI 28.5 ± 5.7 kg/m^2^, 10 055 (82.1%) were males, 8955 (73.1%) were White, 2748 (22.4%) were current smokers, and 7332 (59.9%) had hypertension. Overall median surgical time was 5.3 [4.1–6.8] hours, with 5.2 [4–6.6] hours for patients that underwent RC with iUD and 6.1 [4.6–7.7] hours for those that had RC with cUD (*p* < 0.001) (Table 1).

**TABLE 1:** Clinical and demographic characteristics of the overall population and a comparison between patients in accordance with the type of surgical approach and urinary diversion.

	Overall	Cystectomy with incontinent urinary diversion	Cystectomy with continent urinary diversion	*p*‐value
	*n* = 12 246 (100%)	*n* = 10 308 (84.2%)	*n* = 1938 (15.8%)	
Gender				
Male (%)	10 055 (82.1%)	8371 (81.2%)	1684 (86.9%)	
Female (%)	2190 (17.9%)	1936 (18.8%)	254 (13.1%)	
No binary (%)	1 (0.008%)	1 (0.0%)	0	**<0.001**
Age (years)	69.1 ± 9.6	70.2 ± 9.2	63 ± 9.6	**<0.001**
BMI (kg/m^2^)	28.5 ± 5.7	28.5 ± 5.7	28.6 ± 5.8	0.423
BMI category				
Normal (%)	3127 (25.5%)	2652 (25.7%)	475 (24.5%)	
Underweight (%)	161 (1.3%)	147 (1.4%)	14 (0.7%)	
Overweight (%)	4729 (38.6%)	3944 (38.3%)	785 (40.5%)	
Obese (%)	4152 (33.9%)	3496 (33.9%)	656 (33.8%)	
Unknown (%)	77 (0.6%)	69 (0.7%)	8 (0.4%)	**0.027**
Race/ethnicity				
White (%)	8955 (73.1%)	7505 (72.8%)	1450 (74.8%)	
Black or African American (%)	511 (4.2%)	427 (4.1%)	84 (4.3%)	
Asian (%)	206 (1.7%)	174 (1.7%)	32 (1.7%)	
Other or unknown (%)	2574 (21%)	2202 (21.4%)	372 (19.2%)	0.195
Current smoker within 1 year				
No (%)	9498 (77.6%)	8038 (78%)	1460 (75.3%)	
Smoker (%)	2748 (22.4%)	2270 (22%)	478 (24.7%)	**0.011**
Diabetes mellitus				
No (%)	9861 (80.5%)	8191 (79.5%)	1670 (86.2%)	
Yes (%)	2385 (19.5%)	2117 (20.5%)	268 (13.8%)	**<0.001**
COPD				
No (%)	11 373 (92.9%)	9512 (92.3%)	1861 (96%)	
Yes (%)	873 (7.1%)	796 (7.7%)	77 (4%)	<0.001
Functional status before surgery				
Independent (%)	12 059 (98.5%)	10 130 (98.3%)	1929 (99.5%)	
Partially/totally dependent (%)	156 (1.3%)	149 (1.4%)	7 (0.4%)	
Unknown (%)	31 (0.3%)	29 (0.3%)	2 (0.1%)	**<0.001**
CHF 30 days before surgery				
No (%)	12 157 (99.3%)	10 225 (99.2%)	1932 (99.7%)	
Yes (%)	89 (0.7%)	83 (0.8%)	6 (0.3%)	**0.018**
Hypertension				
No (%)	4914 (40.1%)	3905 (37.9%)	1009 (52.1%)	
Yes (%)	7332 (59.9%)	6403 (62.1%)	929 (47.9%)	**<0.001**
On dialysis before surgery				
No (%)	12 195 (99.6%)	10 262 (99.6%)	1933 (99.7%)	
Yes (%)	51 (0.4%)	46 (0.4%)	5 (0.3%)	0.238
Surgical time (hours)	5.3 [4.1–6.8]	5.2 [4–6.6]	6.1 [4.6–7.7]	**<0.001**
MACE				
No (%)	11 617 (94.9%)	9773 (94.8%)	1844 (95.1%)	
MACE (%)	629 (5.1%)	535 (5.2%)	94 (4.9%)	0.534
Length of stay (days)	7 [5–9]	7 [5–9]	7 [5–9]	0.825

*Note*: Mean ± standard deviation, median [Interquartile range 25th to 75th]. Statistically significant values are presented in bold.

Abbreviations: BMI, body mass index; CHF, congestive heart failure; COPD, chronic obstructive pulmonary disease; MACE, major adverse cardiac events.

A total of 629 (5.1%) patients recorded a MACE, including 56 patients with CVA (45 CVA and 11 combined events), 195 MI (174 MI and 21 combined events), and 407 thromboembolic events (382 thromboembolic events and 25 combined events) (Table S1). On univariable analysis, patients that had MACE were older, had a higher BMI, and had a higher prevalence of comorbidities such as diabetes mellitus, congestive heart failure (CHF) 30 days before surgery, and hypertension. Notably, although the operative time was longer in patients that developed MACE (5.4 [4.1–6.9] hours), compared to those who did not develop MACE (5.3 [4.1–6.8] hours), statistical significance was not reached (*p* = 0.558). There was no difference in the frequency of MACE based on iUD and cUD approach (*p* = 0.534) (Table 2).

**TABLE 2:** Clinical and demographic characteristics of the overall population and a comparison between patients in accordance with the patients that did not developed MACE and those that developed MACE.

	No MACE	MACE	*p*‐value
	*n* = 11 617 (94.9%)	*n* = 629 (5.1%)	
Gender			
Male (%)	9546 (82.2%)	509 (80.9%)	
Female (%)	2070 (17.8%)	120 (19.1%)	
No binary (%)	1 (0.0001%)	0	0.706
Age (years)	69 ± 9.6	71.2 ± 9.4	**<0.001**
BMI (kg/m^2^)	28.5 ± 5.7	29.5 ± 6.1	**<0.001**
BMI category			
Normal (%)	2990 (25.7%)	137 (21.8%)	
Underweight (%)	152 (1.3%)	9 (1.4%)	
Overweight (%)	4522 (38.9%)	207 (32.9%)	
Obese (%)	3882 (33.4%)	270 (42.9%)	
Unknown (%)	71 (0.6%)	6 (1%)	**<0.001**
Race/ethnicity			
White (%)	8521 (73.3%)	434 (69%)	
Black or African American (%)	482 (4.1%)	29 (4.6%)	
Asian (%)	201 (1.7%)	5 (0.8%)	
Other or unknown (%)	2413 (20.8%)	161 (25.6%)	**0.009**
Current smoker within 1 year			
No (%)	9020 (77.6%)	478 (76%)	
Smoker (%)	2597 (22.4%)	151 (24%)	0.334
Diabetes mellitus			
No (%)	9377 (80.7%)	484 (76.9%)	
Yes (%)	2240 (19.3%)	145 (23.1%)	**0.020**
COPD			
No (%)	10 805 (93.0%)	568 (90.3%)	
Yes (%)	812 (7%)	61 (9.7%)	**0.010**
Functional status before surgery			
Independent (%)	11 439 (98.5%)	620 (98.6%)	
Partially/totally dependent (%)	147 (1.3%)	9 (1.4%)	
Unknown (%)	31 (0.3%)	0	0.405
CHF 30 days before surgery			
No (%)	11 538 (99.3%)	619 (98.4%)	
Yes (%)	79 (0.7%)	10 (1.6%)	**0.009**
Hypertension			
No (%)	4713 (40.6%)	201 (32%)	
Yes (%)	6904 (59.4%)	428 (68%)	**<0.001**
On dialysis before surgery			
No (%)	11 570 (99.6%)	625 (99.4%)	
Yes (%)	47 (0.4%)	4 (0.6%)	0.380
Surgical time (hours)	5.3 [4.1–6.8]	5.4 [4.1–6.9]	0.558
Surgical approach			
Cystectomy with incontinent urinary diversion (%)	9773 (84.1%)	535 (85.1%)	
Cystectomy with continent urinary diversion (%)	1844 (15.9%)	94 (14.9%)	0.534
Length of stay (days)	6 [5–9]	9 [6–15]	**<0.001**

*Note*: Mean ± standard deviation, median [Interquartile range 25th to 75th]. Statistically significant values are presented in bold.

Abbreviations: BMI, body mass index; CHF, congestive heart failure; COPD, chronic obstructive pulmonary disease; MACE, major adverse cardiac events.

On the multivariable‐adjusted logistic regression, it was shown that MACE is significantly associated with increased age (OR = 1.035, 95% CI: 1.024–1.046, *p* < 0.001), obesity (OR = 1.583, 95% CI: 1.266–1.978, *p* < 0.001), current smokers (OR = 1.386, 95% CI: 1.130–1.700, *p* = 0.002), CHF before surgery (OR = 1.991, 95% CI: 1.016–3.900; *p* = 0.045), hypertension (OR = 1.209, 95% CI: 1.016–1.453, *p* = 0.043), and increase the surgical time (per 10 min increase, OR = 1.010, 95% CI: 1.003–1.017, *p* = 0.009) (Table 3).

**TABLE 3:** Multivariable adjusted logistic regression analysis reporting the association between 30‐day postoperatively MACE and clinical and demographic characteristics.

	OR	95% CI	*p*‐value
Gender			
Male	Ref		
Female	1.073	0.871–1.322	0.508
Age (per 1 year)	1.035	1.024–1.046	**<0.001**
BMI category			
Normal	Ref		
Underweight	1.250	0.620–2.518	0.533
Overweight	1.005	0.803–1.258	0.963
Obese	1.583	1.266–1.978	**<0.001**
Race/ethnicity			
White	Ref		
Black or African American	1.205	0.813–1.788	0.353
Asian	0.571	0.233–1.401	0.221
Other or unknown	1.390	1.147–1.684	**<0.001**
Current smoker within 1 year			
No	Ref		
Yes	1.386	1.130–1.700	**0.002**
Diabetes mellitus			
No	Ref		
Yes	1.082	0.887–1.321	0.436
COPD			
No	Ref		
Yes	1.252	0.945–1.658	0.117
Functional status before surgery			
Independent	Ref		
Partially/totally dependent	1.166	0.589–2.310	0.660
CHF 30 days before surgery			
No	Ref		
Yes	1.991	1.016–3.900	**0.045**
Hypertension			
No	Ref		
Yes	1.209	1.006–1.453	**0.043**
On dialysis before surgery			
No	Ref		
Yes	1.587	0.562–4.480	0.383
Surgical approach			
Cystectomy with incontinent urinary diversion	Ref		
Cystectomy with continent urinary diversion	1.182	0.932–1.500	0.167
Operative time (per 10 min)	1.010	1.003–1.017	**0.009**

*Note*: Statistically significant values are presented in bold.

Abbreviations: BMI, body mass index; CHF, congestive heart failure; COPD, chronic obstructive pulmonary disease; OR, odds ratio; 95% CI, 95% confidence interval.

**FIGURE 1:**
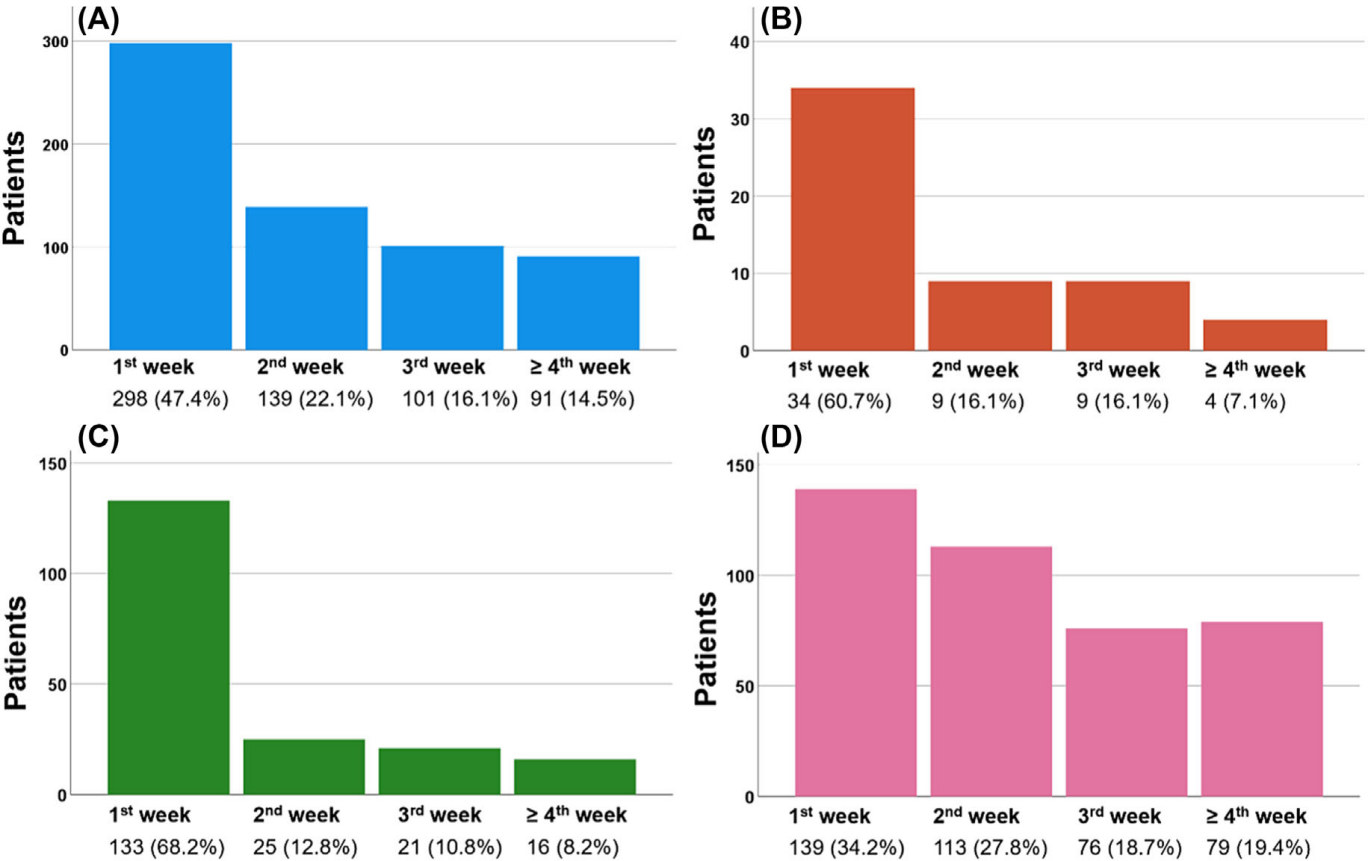
Week in which the MACE was reported: (A) overall MACE, (B) CVA, (C) myocardial infarction, and (D) thromboembolic events.

On multivariable analysis, we found that increased age, current smokers, and CHF were associated with an increased risk of MI. We also report that increased age, obesity, and patients undergoing cUD (OR = 1.368, 95% CI: 1.040–1.798; *p* = 0.025) are associated with thromboembolic events. The increased risk of MACE associated with increased surgical time appears to be driven by the significantly higher risk of thromboembolic events (per 10 min increase, OR = 1.013, 95% CI: 1.004–1.022; *p* = 0.004) (Tables [Link], [Link]). Subset analysis to determine the timing of overall MACE, CVA, MI, and thromboembolic events demonstrated that the incidence was highest during the first postoperative week and declined in each subsequent week over a 30‐day period (Figure 1). We found that 47.4% of MACE occurred within the first postoperative week. Notably, 38.1% of patients who reported a thromboembolic event experienced it following the second postoperative week.

## 4 DISCUSSION

While previous literature has estimated that MACE occurs in 1.4%–4.1% of patients undergoing RC,^10^ our study reported a slightly higher estimate at 5.1%. Overall, thromboembolic events were the most common type of MACE, followed by MI and CVA. Given that longer surgery time is associated with an increased risk of venous thromboembolism, this finding is expected.^11^

We observed differences in the reported rates of thromboembolic events between the RC with cUD versus iUD group. When analysing patients based on median surgical time, we found that creating a cUD requires an additional hour compared to forming an iUD. This finding may stem from the fact that creating a cUD is more technically challenging than forming an iUD.^7^ Surgical and anatomic constraints that require extended operative time may also increase the risk of thromboembolic events and, subsequently, overall MACE. For example, compression of iliac vessels while creating a neobladder or performing a difficult lymph node dissection may lead to thrombosis. This may result in extreme fluid and electrolyte shifts and subsequently cause acute changes in blood pressure and physiologic stress on the myocardium.^13^ High rates of MACE were observed over the first 30‐day postoperative window, with 47.4% of the events occurring within the first postoperative week. This suggests that urologists may be able to avoid the increased risk of MACE by performing RC with iUD for patients in whom RC may be technically more challenging (i.e., prior abdominal surgeries, obesity, and narrow pelvis) and require longer operative time. In addition, limiting RC with cUD to high‐volume centres with extensive expertise may help to keep operative times low and limit the risk of overall MACE.^13^

In this study, we found that age, obesity, smoking, CHF, and hypertension were associated with an increased risk of MACE following RC. Prior studies have shown that patients older than 65 years have a higher risk of MACE for one‐, two‐, and three‐vessel diseases when compared to individuals less than 65 years.^14^ This is particularly relevant to our study because the average age for patients who developed MACE was 71.2, and a 1‐year increase in age was associated with a 3.5% increase in reported MACE. Our sample reflects a selection bias for younger patients who undergo a cUD approach due to the assumption that they may be better able to withstand an intensive procedure. However, we observed the highest rate of thromboembolic events in patients undergoing cUD. This suggests that the protective benefit of younger age in cUD may be offset when longer operative time is required. The iROC trial (robot‐assisted radical cystectomy with intracorporeal urinary diversion versus open radical cystectomy) showed patients ≥75 years and older who underwent a robotic approach have shorter hospital stay than those who underwent open RC.^15^ However, our study suggests that there is an increased incidence in postoperative MACE in elderly patients. Further studies are needed to compare MACE rates in elderly patients based on surgical approach.

Our finding of an increased risk of MACE with increasing BMI and in those who are current smokers has also been well documented in the literature. It has been shown that increased abdominal obesity is associated with an elevated risk of major cardiovascular outcomes.^16^ Additionally, previous studies have reported a 1.7 times higher risk of MI and a 1.6 times higher risk of CVA in smokers, compared to non‐smokers.^17^ These findings highlight the importance for surgeons to evaluate the pre‐operative characteristics of patients when selecting a urinary diversion approach to decrease the incidence of MACE in the post‐operative setting. In addition, studies have shown that hypertension is an important precursor of cardiovascular disease in the elderly.^18^ We found that 68% of patients with MACE were hypertensive, compared to 60% of patients without MACE.

Approximately 38.1% of thromboembolic events occurred during or following postoperative week 3 in this cohort. Previous evidence from major abdominal, pelvic, and oncologic surgery cases suggests that there is a benefit for the use of thromboembolic prophylaxis in patients undergoing RC.^19^, ^20^, ^21^, ^22^ A meta‐analysis that used randomized and prospective studies with abdominal or pelvic surgery for cancer found that extended thromboprophylaxis was associated with reduced incidence of all VTEs (2.6 vs. 5.6%; relative risk = 0.44) and reduced incidence of proximal DVT (1.4 vs. 2.8%; relative risk = 0.46).^23^ Our findings similarly support the use of extended thromboembolic prophylaxis following RC. Furthermore, the use of thromboembolic prophylaxis beyond 4 weeks may potentially be considered in patients with the observed risk factors including older age, current smoking status, obesity, and hypertension or those who underwent a prolonged RC with cUD. Future studies assessing the use of prolonged thromboembolic prophylaxis in patients undergoing RC and following risk stratification would be of clinical value.

Limitations of this study include the use of a retrospective database, which may lack the granular data needed to accurately assess causal factors of MACE. Due to restrictions in data accessibility, we were unable to acquire information on the technical skills of surgeons, additional information on patients' pre‐operative conditions (e.g., history of atrial fibrillation), perioperative medical management (e.g., use of antibiotics prophylaxis or DVT prophylaxis), surgical approach (open, laparoscopic, or robotic‐assisted), surgical technique for urinary diversion (intracorporeal or extracorporeal), incidence of postoperative metabolic complications, or Clavien‐Dindo classification of surgical complications. Additionally, we were unable to assess whether patients received neoadjuvant chemotherapy, which is known to increase VTE risk. It would be useful to assess whether patients received heparin perioperatively and/or continued enoxaparin or direct oral anticoagulant at home, and the length of time after surgery in which these medications were prescribed. Additionally, we could not access detailed information on postoperative MACE (e.g., type of stroke or extension of DVT) to consider this information within the analysis. Strengths of this study include the large sample size and multi‐centre data, along with specific information regarding complications in the first postoperative month, which adds to the current knowledge regarding risk factors associated with developing MACE after undergoing RC. We believe that further efforts should aim to create a scoring system that assesses the clinical and surgical factors associated with MACE and provides recommendations for the perioperative management of patients in accordance with their MACE risk group, including the length of postoperative DVT prophylaxis. By utilizing data from multiple surgeons from different institutions, a comprehensive scoring system may be developed.

By considering the preoperative characteristics of patients, including age, obesity, smoking, congestive heart failure, and hypertension status, urologists may be able to recognize patients with a higher risk of postoperative MACE and potentially decrease the incidence of MACE in patients undergoing RC by modifying the perioperative management of higher risk patients. In addition, our findings assist clinicians with preoperative counselling and ensuring medical optimization for patients at risk. Urologists should aim for lower operative times, as this was associated with a decreased risk of thromboembolic events. Further prospective studies need to be performed to confirm our findings.

## AUTHOR CONTRIBUTIONS

**Aaron A. Gurayah**: Conceptualization; methodology; data curation; formal analysis; writing—original draft. **Ruben Blachman‐Braun**: Conceptualization; methodology; data curation; formal analysis; writing—original draft; writing—review and editing. **Christopher J. Machado**: Writing—original draft. **Matthew M. Mason**: Writing—original draft. **Helen Y. Hougen**: Writing—review and editing. **Ali Mouzannar**: Writing—review and editing. **Mark L. Gonzalgo**: Writing—review and editing. **Bruno Nahar**: Writing—review and editing. **Sanoj Punnen**: Writing—review and editing. **Dipen J. Parekh**: Writing—review and editing. **Chad R. Ritch**: Conceptualization; investigation; methodology; writing—review and editing.

## CONFLICT OF INTEREST STATEMENT

The authors have no conflict of interest to disclose.

## Supporting information


**Figure S1.** Consort diagram showing selection criteria for all patients.

**Table S1.** Frequency of reported MACE.

**Table S2.** Multivariable adjusted logistic regression analysis reporting the association between 30‐days postoperatively CVA and clinical and demographic characteristics.

**Table S3.** Multivariable adjusted logistic regression analysis reporting the association between 30‐days postoperatively myocardial infraction and clinical and demographic characteristics.

**Table S4.** Multivariable adjusted logistic regression analysis reporting the association between 30‐days postoperatively thromboembolic event and clinical and demographic characteristics.
