# Supplementary figures and images for: Clinical variables associated with major adverse cardiac events following radical cystectomy
Source: BJUI Compass. 2023 Dec 5;5(4):480–8. doi: 10.1002/bco2.315 (PMC11019239; doi:10.1002/bco2.315)

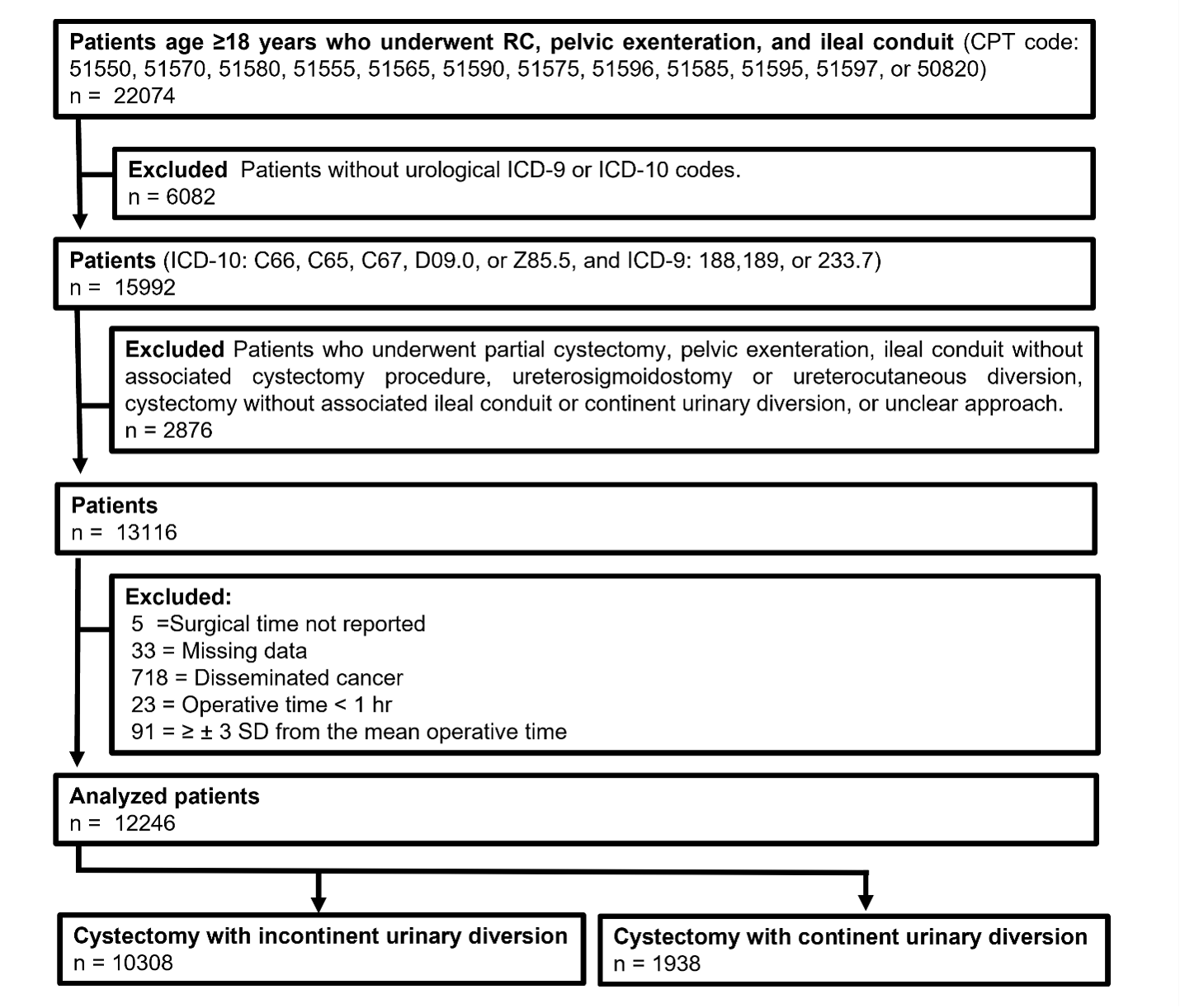

Supplement: Supplementary file 1 — Figure S1. Consort diagram showing selection criteria for all patients. [file BCO2-5-480-s004.png]
