# Supplementary material for: Clinical variables associated with major adverse cardiac events following radical cystectomy
Source: BJUI Compass. 2023 Dec 5;5(4):480–8. doi: 10.1002/bco2.315 (PMC11019239; doi:10.1002/bco2.315)
Supplement: Supplementary file 2 — Table S1. Frequency of reported MACE. [file BCO2-5-480-s001.docx]

**Supplementary table 1**. Frequency of reported MACE.

|  | **Type of MACE** |
| --- | --- |
| **Thromboembolic events** (%) | 382 (60.7%) |
| **Myocardial infarction** (%) | 174 (27.7%) |
| **CVA** (%) | 45 (7.2%) |
| **Thromboembolic events and myocardial infarction** (%) | 17 (2.7%) |
| **CVA and thromboembolic events** (%) | 7 (1.1%) |
| **CVA and myocardial infarction** (%) | 3 (0.5%) |
| **CVA, thromboembolic events and myocardial infarction** (%) | 1 (0.2%) |

CVA: Cerebrovascular accident, MACE: Major adverse cardiac events.
