# Supplementary material for: Clinical variables associated with major adverse cardiac events following radical cystectomy
Source: BJUI Compass. 2023 Dec 5;5(4):480–8. doi: 10.1002/bco2.315 (PMC11019239; doi:10.1002/bco2.315)
Supplement: Supplementary file 3 — Table S2. Multivariable adjusted logistic regression analysis reporting the association between 30‐days postoperatively CVA and clinical and demographic characteristics. [file BCO2-5-480-s003.docx]

**Supplementary table 2.** Multivariable adjusted logistic regression analysis reporting the association between 30-days postoperatively CVA and clinical and demographic characteristics.

|  | **OR** | **95% CI** | **P-value** |
| --- | --- | --- | --- |
| **Gender** |  |  |  |
| Male | Ref |  |  |
| Female | 1.201 | 0.626 - 2.307 | 0.582 |
| **Age** (per 1 years) | 1.030 | 0.995 - 1.066 | 0.094 |
| **BMI category** |  |  |  |
| Normal | Ref |  |  |
| Underweight | 2.589 | 0.573 - 11.711 | 0.217 |
| Overweight | 1.092 | 0.555 - 2.148 | 0.799 |
| Obese | 0.920 | 0.437 - 1.935 | 0.825 |
| **Race / ethnicity** |  |  |  |
| White | Ref |  |  |
| Black or African American | 1.313 | 0.396 - 4.354 | 0.656 |
| Other or unknown | 1.532 | 0.838 - 2.799 | 0.166 |
| **Current smoker within one year** |  |  |  |
| No | Ref |  |  |
| Yes | 1.643 | 0.868 - 3.109 | 0.127 |
| **Diabetes mellitus** |  |  |  |
| No | Ref |  |  |
| Yes | 1.101 | 0.579 - 2.096 | 0.769 |
| **COPD** |  |  |  |
| No | Ref |  |  |
| Yes | 1.238 | 0.517 - 2.963 | 0.631 |
| **Functional status before surgery** |  |  |  |
| Independent | Ref |  |  |
| Partially / Totally Dependent | 1.430 | 0.195 - 10.467 | 0.725 |
| **Hypertension** |  |  |  |
| No | Ref |  |  |
| Yes | 1.853 | 0.978 - 3.511 | 0.058 |
| **On dialysis before surgery** |  |  |  |
| No | Ref |  |  |
| Yes | 4.557 | 0.601 - 34.569 | 0.142 |
| **Surgical approach** |  |  |  |
| Cystectomy with incontinent urinary diversion | Ref |  |  |
| Cystectomy with continent urinary diversion | 0.552 | 0.194 - 1.570 | 0.265 |
| **Surgical time** (per 10 minutes) | 1.001 | 0.976 - 1.026 | 0.935 |

BMI: Body mass index, CHF: Congestive heart failure, COPD: Chronic obstructive pulmonary disease, OR: Odds ratio, 95% CI: 95% confidence interval.
