# Supplementary material for: Clinical variables associated with major adverse cardiac events following radical cystectomy
Source: BJUI Compass. 2023 Dec 5;5(4):480–8. doi: 10.1002/bco2.315 (PMC11019239; doi:10.1002/bco2.315)
Supplement: Supplementary file 4 — Table S3. Multivariable adjusted logistic regression analysis reporting the association between 30‐days postoperatively myocardial infraction and clinical and demographic characteristics. [file BCO2-5-480-s005.docx]

**Supplementary table 3.** Multivariable adjusted logistic regression analysis reporting the association between 30-days postoperatively myocardial infraction and clinical and demographic characteristics.

|  | **OR** | **95% CI** | **P-value** |
| --- | --- | --- | --- |
| **Gender** |  |  |  |
| Male | Ref |  |  |
| Female | 0.770 | 0.512 - 1.159 | 0.211 |
| **Age** (per 1 years) | 1.060 | 1.039 - 1.081 | **< 0.001** |
| **BMI category** |  |  |  |
| Normal | Ref |  |  |
| Underweight | 0.381 | 0.052 - 2.796 | 0.343 |
| Overweight | 0.840 | 0.577 - 1.223 | 0.363 |
| Obese | 1.197 | 0.818 - 1.751 | 0.355 |
| **Race / ethnicity** |  |  |  |
| White | Ref |  |  |
| Black or African American | 0.746 | 0.301 - 1.851 | 0.528 |
| Asian | 0.757 | 0.184 - 3.110 | 0.700 |
| Other or unknown | 1.757 | 1.277 - 2.416 | **< 0.001** |
| **Current smoker within one year** |  |  |  |
| No | Ref |  |  |
| Yes | 1.584 | 1.102 - 2.277 | **0.013** |
| **Diabetes mellitus** |  |  |  |
| No | Ref |  |  |
| Yes | 1.268 | 0.908 - 1.7690 | 0.163 |
| **COPD** |  |  |  |
| No | Ref |  |  |
| Yes | 1.264 | 0.789 - 2.023 | 0.330 |
| **Functional status before surgery** |  |  |  |
| Independent | Ref |  |  |
| Partially / Totally Dependent | 1.624 | 0.589 - 4.476 | 0.349 |
| **CHF 30 days before surgery** |  |  |  |
| No | Ref |  |  |
| Yes | 3.679 | 1.556 - 8.697 | **0.003** |
| **Hypertension** |  |  |  |
| No | Ref |  |  |
| Yes | 1.385 | 0.990 - 1.937 | 0.057 |
| **On dialysis before surgery** |  |  |  |
| No | Ref |  |  |
| Yes | 2.391 | 0.561 - 10.185 | 0.238 |
| **Surgical approach** |  |  |  |
| Cystectomy with incontinent urinary diversion | Ref |  |  |
| Cystectomy with continent urinary diversion | 0.929 | 0.573 - 1.508 | 0.767 |
| **Surgical time** (per 10 minutes) | 1.005 | 0.991 - 1.018 | 0.490 |

BMI: Body mass index, CHF: Congestive heart failure, COPD: Chronic obstructive pulmonary disease, OR: Odds ratio, 95% CI: 95% confidence interval.
