# Supplementary material for: Clinical variables associated with major adverse cardiac events following radical cystectomy
Source: BJUI Compass. 2023 Dec 5;5(4):480–8. doi: 10.1002/bco2.315 (PMC11019239; doi:10.1002/bco2.315)
Supplement: Supplementary file 5 — Table S4. Multivariable adjusted logistic regression analysis reporting the association between 30‐days postoperatively thromboembolic event and clinical and demographic characteristics. [file BCO2-5-480-s002.docx]

**Supplementary table 4.** Multivariable adjusted logistic regression analysis reporting the association between 30-days postoperatively thromboembolic event and clinical and demographic characteristics.

|  | **OR** | **95% CI** | **P-value** |
| --- | --- | --- | --- |
| **Gender** |  |  |  |
| Male | Ref |  |  |
| Female | 1.198 | 0.935 - 1.535 | 0.153 |
| **Age** (per 1 years) | 1.027 | 1.014 - 1.039 | **<0.001** |
| **BMI category** |  |  |  |
| Normal | Ref |  |  |
| Underweight | 1.677 | 0.756 - 3.720 | 0.204 |
| Overweight | 1.130 | 0.847 - 1.507 | 0.407 |
| Obese | 1.948 | 1.470 - 2.583 | **<0.001** |
| **Race / ethnicity** |  |  |  |
| White | Ref |  |  |
| Black or African American | 1.587 | 1.035 - 2.435 | 0.034 |
| Asian | 0.555 | 0.176 - 1.755 | 0.316 |
| Other or unknown | 1.215 | 0.951 - 1.551 | 0.119 |
| **Current smoker within one year** |  |  |  |
| No | Ref |  |  |
| Yes | 1.221 | 0.949 - 1.573 | 0.121 |
| **Diabetes mellitus** |  |  |  |
| No | Ref |  |  |
| Yes | 0.929 | 0.719 - 1.200 | 0.571 |
| **COPD** |  |  |  |
| No | Ref |  |  |
| Yes | 1.289 | 0.908 - 1.828 | 0.155 |
| **Functional status before surgery** |  |  |  |
| Independent | Ref |  |  |
| Partially / Totally Dependent | 0.796 | 0.292 - 2.168 | 0.655 |
| **CHF 30 days before surgery** |  |  |  |
| No | Ref |  |  |
| Yes | 1.224 | 0.443 - 3.381 | 0.697 |
| **Hypertension** |  |  |  |
| No | Ref |  |  |
| Yes | 1.037 | 0.831 - 1.294 | 0.747 |
| **On dialysis before surgery** |  |  |  |
| No | Ref |  |  |
| Yes | 0.595 | 0.081 - 4.351 | 0.609 |
| **Surgical approach** |  |  |  |
| Cystectomy with incontinent urinary diversion | Ref |  |  |
| Cystectomy with continent urinary diversion | 1.368 | 1.040 - 1.798 | **0.025** |
| **Surgical time** (per 10 minutes) | 1.013 | 1.004 - 1.022 | **0.004** |

BMI: Body mass index, CHF: Congestive heart failure, COPD: Chronic obstructive pulmonary disease, OR: Odds ratio, 95% CI: 95% confidence interval.
